# Isolated Gastric Variceal Bleeding: A Sentinel Sign of Pancreatic Neoplasm Manifesting as Sinistral Portal Hypertension

**DOI:** 10.7759/cureus.100992

**Published:** 2026-01-07

**Authors:** Sergio David Angulo, Julian Toro, Jesús Hinestroza, Isabel Sánchez, David Cataño, Leisly T Oviedo Gomez

**Affiliations:** 1 Research and Teaching Unit, Clinica Comfamiliar, Manizales, COL; 2 Research and Teaching Unit, Clinica Comfamiliar, Pereira, COL; 3 Research and Teaching Unit, Clinica Comfamiliar, Risaralda, COL; 4 Internal Medicine Department, Clinica Comfamiliar, Pereira, COL; 5 Psychiatry Department, Clínica Unión Mental, Ibague, COL

**Keywords:** case report, gastric varices, left-sided portal hypertension, pancreatic cancer, sinistral portal hypertension

## Abstract

Sinistral portal hypertension (SPH) is an uncommon condition secondary to stenosis of the splenic vein. The most common causes are chronic pancreatitis and pancreatic cancer. This localized hypertension can lead to the development of isolated gastric varices (IGV) in non-cirrhotic patients and potentially life-threatening upper gastrointestinal bleeding. We report a case of pancreatic cancer causing left-sided portal hypertension (LSPH) and isolated gastric varices bleeding. Pancreatic cancer is one of the leading causes of cancer-related death globally. Symptoms are non-specific, and there are no routine screening tests; therefore, the disease tends to be diagnosed in advanced stages. A high suspicion index and systematic diagnosis approach are needed, including inflammatory, infiltrative, and oncological causes, to establish a proper diagnosis.

## Introduction

Sinistral portal hypertension (SPH), also known as left-sided portal hypertension (LSPH), segmental, regional, or splenoportal hypertension, is a rare clinical syndrome resulting from the obstruction or stenosis of the splenic vein [1]. The most common underlying causes are pancreatic diseases, including chronic pancreatitis, pancreatic pseudocysts, and pancreatic neoplasms [2]. This condition can lead to the formation of isolated gastric varices (IGV) and upper gastrointestinal bleeding in non-cirrhotic patients [3]. IGV are uncommon, accounting for only 4.7% of all gastric varices in patients with portal hypertension [4]. IGV are classified endoscopically using Sarin's classification, which is based on the varix's anatomical location [5]. IGV type 1 (IGV1) are located in the fundus, and IGV type 2 are distal gastric varices or those found in other sporadic locations [6]. We present a case report of SPH presenting as the initial manifestation of a pancreatic neoplasm. This case report has been reported in line with the CARE checklist [7].

## Case presentation

**Figure 1:**
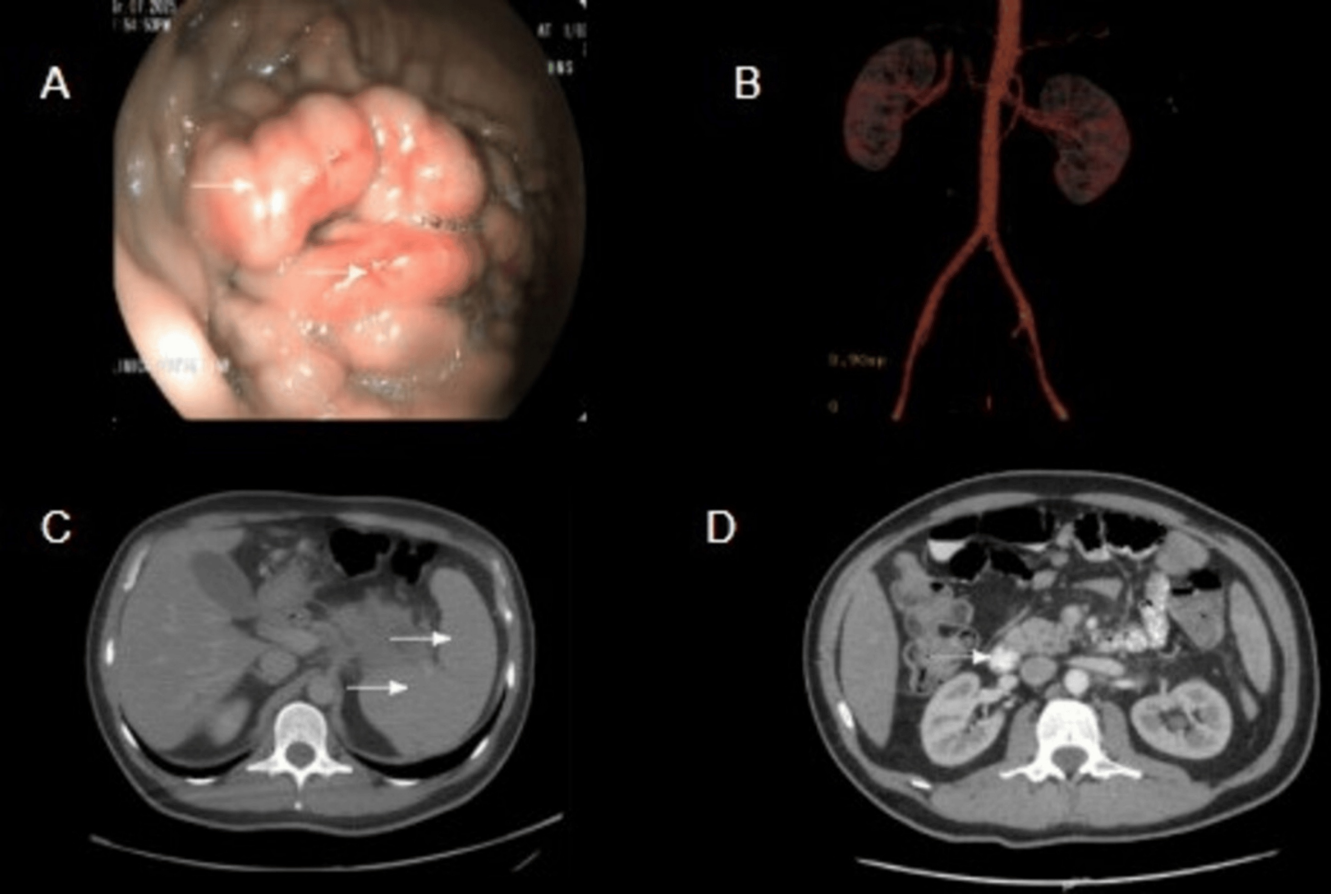
Multimodal imaging findings (A) EGD: arrows showing IGV1. (B) Angiography demonstrating a large gastrorenal shunt (outflow) directed toward the left renal vein. (C) Abdominal enhanced CT scan in portal venous phase: arrows showing dilated portal vein and splenomegaly. (D) Abdominal enhanced CT: arrow showing a hypodense pancreatic lesion. EGD: esophagogastroduodenoscopy, IGV1: isolated gastric varices type 1, CT: computed tomography

A previously healthy 48-year-old man presented to the emergency department complaining of epigastric abdominal pain, hematemesis, and occasional melena. On admission, he exhibited hypovolemia, tachycardia, and pale oral mucosa. Notably, physical examination showed no external stigmata of chronic liver disease. Initial management for presumed upper gastrointestinal bleeding was instituted, including intravenous fluid resuscitation, analgesics, and proton pump inhibitors (PPIs). At this stage, differential diagnoses included peptic ulcer disease, Mallory-Weiss tear, and variceal bleeding. A comprehensive diagnostic workup was ordered, comprising a complete blood count (CBC), renal function tests, hepatobiliary profile, and abdominal ultrasound (Table 1). An urgent esophagogastroduodenoscopy (EGD) was performed on the next day, identifying isolated gastric varices type 1 (IGV1) as the source of the hemorrhage (Figure 1A).

**Table 1:** Laboratory data at admission AST: aspartate aminotransferase, ALT: alanine aminotransferase, aPTT: activated partial thromboplastin time, INR: international normalized ratio

Parameter	Patient value	Reference range
Hemoglobin (gr/L)	10.1	11.4-14.7
Mean corpuscular volume (fL)	85.9	80-100
Hematocrit (%)	30.5	36.9-54.3
Red cell distribution width (%)	12.1	12-16
White blood cells (cells/μL)	7,840	3,500-13,500
Neutrophils (cells/μL)	5,820	2,500-8,000
Eosinophils (cells/μL)	0.09	0-330
Lymphocytes (cells/μL)	1,400	1,000-4,800
Platelet count (cells/μL)	147,000	150,000-450,000
Creatinine (mg/dL)	0.99	1.1
Blood urea nitrogen (mg/dL)	17.67	6-20
AST (UI/L)	14.4	5-34
ALT (UI/L)	11.6	0-55
Total bilirubin (mg/dL)	0.30	0.1-1
Direct bilirubin (mg/dL)	0.13	0.1-0.3
Indirect bilirubin (mg/dL)	0.17	0.2-0.7
Alkaline phosphatase (UI/L)	58.50	50-116
Amylase (UI/L)	38.9	28-100
Prothrombin time (seconds)	11.4	9.9-11.8
aPTT (seconds)	23.1	25-31.3
INR	1.08	0-1.1

Initial investigation via abdominal ultrasound yielded conflicting results; it reported a patent portal vein with normal hepatopetal flow and a spleen of normal size, effectively ruling out typical signs of generalized portal hypertension. However, given the critical endoscopic finding of IGV1 standing in stark contrast to the unremarkable ultrasound, the case was discussed with the interventional radiology team. An abdominal computed tomography (CT) angiography was requested, revealing portal hypertension with a large gastrorenal shunt that was successfully embolized without complications (Figure 1B).

Despite effective control of the acute bleeding, a clear etiology for the portal hypertension remained unknown after the initial exhaustive workup (Figure 1C). Consequently, the interdisciplinary team proceeded with advanced imaging. A contrast-enhanced abdominal CT radically shifted the diagnostic focus and identified a primary hyperdense mass involving the body and tail of the pancreas, with retroperitoneal lymphadenopathy (Figure 1D). This finding was accompanied by abnormally high CA 19-9 levels (926 UI/mL). The abdominal CT also revealed signs of diffuse liver cirrhosis with associated cysts and nodules.

Before confirming the pancreatic etiology, the causes of chronic liver disease were ruled out. Viral hepatitis serologies, autoimmune profiles, and workup for Wilson's disease returned negative results (Table 2). Additional testing, notably a pancreatic biopsy, ultimately revealed pancreatic adenocarcinoma. The patient is currently undergoing chemotherapy and shows good clinical progress.

**Table 2:** Further laboratory testing ANA: antinuclear antibody, ANCA: antineutrophil cytoplasmic antibody, ASMA: anti-smooth-muscle antibody

Parameter	Patient value	Reference range
Ceruloplasmin (mg/dL)	10	20-40
ANA	2:20	<1:80
ANCA	Negative	Negative
ASMA (U)	Negative	>30 U
Reticulocyte count (%)	2.9	0.88-2.37
Peripheral blood smear	Hypochromia (+), mild anisocytosis microcytes (+), macrocytes (+), ovalocytes (+)	Normal morphology
Haptoglobin (mg/dL)	173	14-268
Direct Coombs test	Negative	Negative
Transferrin saturation (%)	16	20-50
Serum iron (µg/dL)	34	65-175
Ferritin (ng/mL)	35	21.81-274.66
24-hour urinary copper (ug/24 hours)	14	3-50
Vitamin B12 (pg/mL)	879	187-883
CA 19-9 (U/mL)	926	0-37

## Discussion

Pancreatic cancer is one of the leading causes of cancer-related death globally. It currently ranks as the fifth leading cause of cancer death in the United Kingdom and is projected to climb to the third leading cause of death by 2030 in the United States [8]. The estimated annual incidence of pancreatic cancer in Colombia is approximately 2,812 new cases, representing about 2.4% of the total cancer burden [9]. The clinical course is often challenging because the disease remains asymptomatic until advanced stages. When symptoms emerge, they are usually non-specific, presenting as loss of appetite and changes in bowel habits [10]. Because effective population-level screening methods are lacking, the disease tends to be diagnosed in advanced stages [11].

Pancreatic cancer is a key contributor to SPH [12]. While SPH is a rare condition, accounting for less than 5% of all gastric varices in portal hypertension patients, pancreatic neoplasms, including malignant (pancreatic adenocarcinoma) and benign lesions (pancreatic cyst), are the second most common cause of splenic vein obstruction [13]. A registry of 209 cases of splenic vein obstruction found that pancreatic neoplasm was responsible for approximately 18% of cases, surpassed only by pancreatitis (65%) [14].

**Figure 2:**
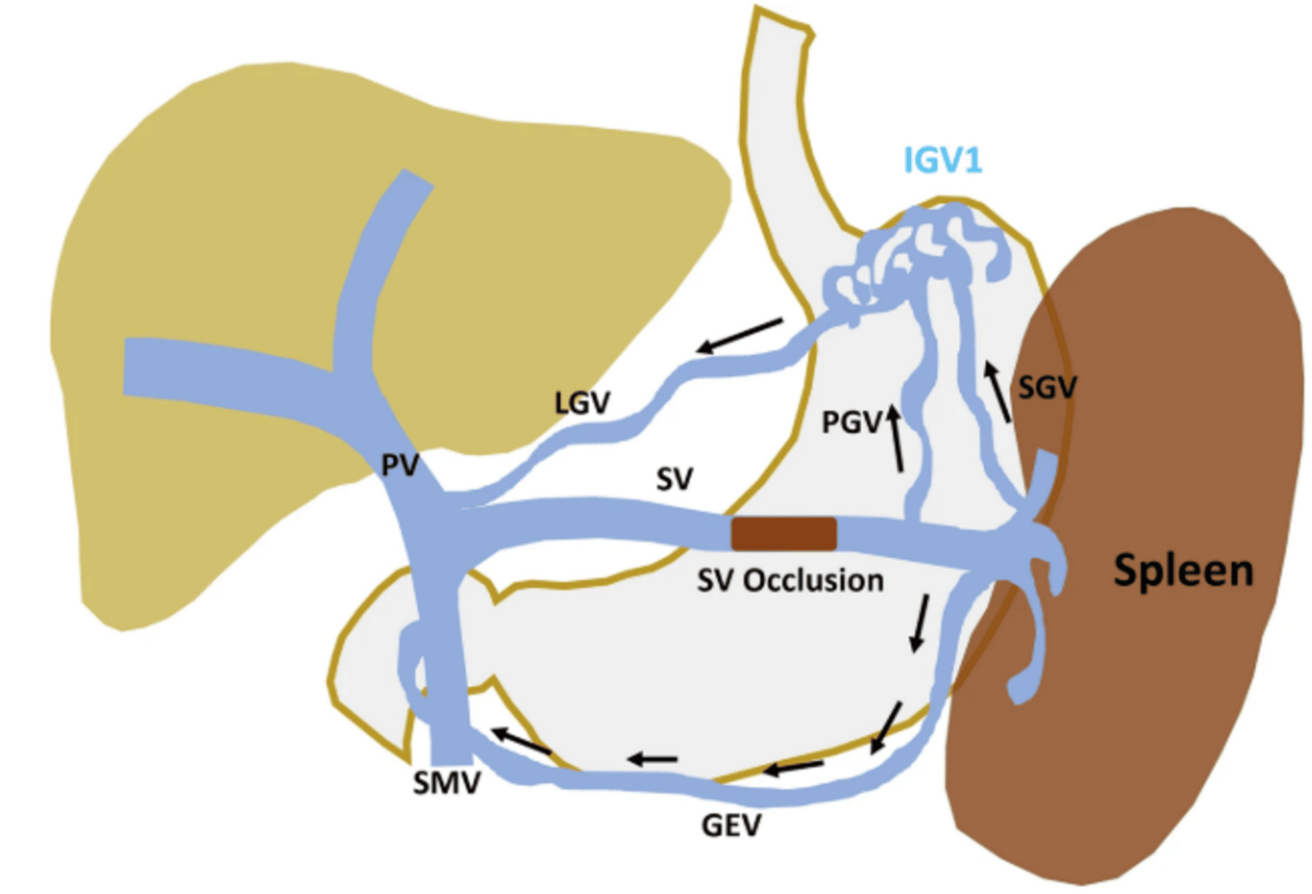
Schematic diagram showing the pathophysiology of LSPH Splenic vein occlusion leads to splenic venous hypertension, which results in the diversion of flow through the SGV, PGV, and GEV. Splenofugal flow in PGV and SGV forms gastric fundal varices and drains into the PV via LGV. GEV also drains splenic venous flow to PV. LSPH: left-sided portal hypertension, SGV: short gastric vein, PGV: posterior gastric vein, GEV: gastroepiploic vein, PV: portal vein, LGV: left gastric vein, SV: splenic vein Adapted with permission from Patel et al. [17]

IGV1 secondary to splenic vein compression results from a distinct hemodynamic mechanism. When the splenic vein is obstructed, the normal venous outflow from the spleen is impeded, leading to retrograde flow through the short gastric veins [15]. These veins, which connect the splenic hilum to the greater curvature of the stomach, become engorged, forming the fundal varices observed endoscopically [16]. To bypass the obstruction, the high-pressure blood flow seeks a systemic escape route, draining from the varices into the inferior phrenic vein or directly creating a gastrorenal shunt, as was clearly visualized in our patient's angiography (Figure 2) [17].

Management of SPH focuses primarily on treating the underlying cause. For definitive treatment of SPH, both open surgical and endovascular procedures have been established. However, in patients who are poor surgical candidates or require immediate stabilization, splenic artery embolization is an effective alternative. It is important to note that transjugular intrahepatic portosystemic shunt (TIPS) is generally not indicated in SPH cases. TIPS leads to direct decompression of the portal pressure (right-sided portal hypertension), which is usually not elevated in cases of SPH (left-sided portal hypertension).

## Conclusions

The identification of IGV1 as the source of upper gastrointestinal bleeding in a patient without external stigmata of chronic liver disease poses a significant diagnostic challenge. The differential diagnosis is broad, encompassing inflammatory, infiltrative, and oncological causes. Therefore, a multidisciplinary and systematic approach is crucial, initially evaluating the most common etiologies before proceeding in a stepwise manner toward more complex diagnoses.

## References

[1] Fernandes A, Almeida N, Ferreira AM (2015). Left-sided portal hypertension: a sinister entity. GE Port J Gastroenterol.

[2] Mayer P, Venkatasamy A, Baumert TF, Habersetzer F, Pessaux P, Saviano A, Felli E (2024). Left-sided portal hypertension: update and proposition of management algorithm. J Visc Surg.

[3] Seijo J, Loarte DV, Martínez M (2004). Left portal hypertension with bleeding from gastric varices as a presentation of hypernephroma (Article in Spanish). An Med Interna (Madrid).

[4] Sompalli S, Faiek S, Mallari M, Camarena J 3rd (2020). Bleeding isolated gastric varices as a rare presentation of pancreatic neuroendocrine tumor: case report and literature review. Cureus.

[5] Sarin SK, Jain AK, Lamba GS, Gupta R, Chowdhary A (2003). Isolated gastric varices: prevalence, clinical relevance and natural history. Dig Surg.

[6] Sarin SK, Lahoti D, Saxena SP, Murthy NS, Makwana UK (1992). Prevalence, classification and natural history of gastric varices: a long-term follow-up study in 568 portal hypertension patients. Hepatology.

[7] Gagnier JJ, Kienle G, Altman DG, Moher D, Sox H, Riley D (2013). The CARE guidelines: consensus-based clinical case reporting guideline development. BMJ Case Rep.

[8] (2025). Cancer Research UK: Pancreatic cancer statistics. https://www.cancerresearchuk.org/health-professional/cancer-statistics/statistics-by-cancer-type/pancreatic-cancer.

[9] Ilic I, Ilic M (2022). International patterns in incidence and mortality trends of pancreatic cancer in the last three decades: a joinpoint regression analysis. World J Gastroenterol.

[10] (2025). Global Cancer Observatory: Cancer Today. https://gco.iarc.who.int/today.

[11] Park W, Chawla A, O'Reilly EM (2021). Pancreatic cancer: a review. JAMA.

[12] Zheng K, Guo X, Feng J (2020). Gastrointestinal bleeding due to pancreatic disease-related portal hypertension. Gastroenterol Res Pract.

[13] Wang L, Liu GJ, Chen YX, Dong HP, Wang LX (2012). Sinistral portal hypertension: clinical features and surgical treatment of chronic splenic vein occlusion. Med Princ Pract.

[14] Maydeo A, Patil G (2022). How to approach a patient with gastric varices. Gastroenterology.

[15] Lupascu-Ursulescu C, Trofin AM, Zabara M (2017). Bleeding from isolated gastric varices as complication of a mucinous cystic neoplasm of the pancreas: a case report. Medicine (Baltimore).

[16] Thrainsdottir H, Petursdottir V, Blöndal S, Björnsson ES (2014). Pancreatic mass leading to left-sided portal hypertension, causing bleeding from isolated gastric varices. Case Rep Gastrointest Med.

[17] Patel RK, Tripathy T, Chandel K (2025). Left-sided portal hypertension: what an interventional radiologist can offer?. Eur Radiol.

